# Saunders’ Question-Compends No. 15

**Published:** 1891-02

**Authors:** 


SAUNDERS' QUESTION-COMPENDS No. 15.—Essentials of the Diseases of Children, Arranged in the form of Questions and Answers, prepared especially for Students of Medicine. By William M. Powell, M. D., Physician to the Clinic for Diseases of Children in the Hospital of the University of Pennsylvania; Examining Physician to the Children's Seashore House for Invalid Children, at Atlantic City, N. J., formerly Instructor in Physical Diagnosis in the Medical Department of the University of Pennsylvania, and Chief of the Medical Clinic of the Philadelphia Polyclinic.

This little work simplifies the subjects treated, and adopts itself to the wants of students of medicine. It is unpretentious, but full of useful information. Saunders' Question'Compends are worthy the attention of the beginners of medicine. They are generally well suited to the use of those who have not time to devote to large works. A. W. G.

